# Depletion of the cellular antioxidant system contributes to tenofovir disoproxil fumarate - induced mitochondrial damage and increased oxido-nitrosative stress in the kidney

**DOI:** 10.1186/1423-0127-20-61

**Published:** 2013-08-19

**Authors:** Premila Abraham, Hemalatha Ramamoorthy, Bina Isaac

**Affiliations:** 1 Department of Biochemistry, Christian Medical College, Bagayam, Vellore 632002, Tamil Nadu, India; 2 Department of Anatomy, Christian Medical College, Bagayam, Vellore 632002, Tamil Nadu India

**Keywords:** Tenofovir, Proximal tubular damage, Mitochondrial damage, Mitochondrial antioxidant system

## Abstract

**Background:**

Nephrotoxicity is a dose limiting side effect of tenofovir, a reverse transcriptase inhibitor that is used for the treatment of HIV infection. The mechanism of tenofovir nephrotoxicity is not clear. Tenofovir is specifically toxic to the proximal convoluted tubules and proximal tubular mitochondria are the targets of tenofovir cytotoxicity. Damaged mitochondria are major sources of reactive oxygen species and cellular damage is reported to occur after the antioxidants are depleted. The purpose of the study is to investigate the alterations in cellular antioxidant system in tenofovir induced renal damage using a rat model.

**Results:**

Chronic tenofovir administration to adult Wistar rats resulted in proximal tubular damage (as evidenced by light microscopy), proximal tubular dysfunction (as shown by Fanconi syndrome and tubular proteinuria), and extensive proximal tubular mitochondrial injury (as revealed by electron microscopy). A 50% increase in protein carbonyl content was observed in the kidneys of TDF treated rats as compared with the control. Reduced glutathione was decreased by 50%. The activity of superoxide dismutase was decreased by 57%, glutathione peroxidase by 45%, and glutathione reductase by 150% as compared with control. Carbonic Anhydrase activity was decreased by 45% in the TDF treated rat kidneys as compared with control. Succinate dehydrogenase activity, an indicator of mitochondrial activity was decreased by 29% in the TDF treated rat kidneys as compared with controls, suggesting mitochondrial dysfunction.

**Conclusion:**

Tenofovir- induced mitochondrial damage and increased oxidative stress in the rat kidneys may be due to depletion of the antioxidant system particularly, the glutathione dependent system and MnSOD.

## Background

Tenofovir disoproxil fumarate (TDF) is an oral prodrug of tenofovir, a reverse transcriptase inhibitor of human immunodeficiency virus type 1 (HIV-1). It is currently the only NRTI (nucleotide analogue reverse-transcriptase inhibitor) that is approved by the Food and Drug administration (FDA), USA for the treatment of HIV infection [1,2]. However, TDF has serious side effects, especially with long-term use. In recent days renal toxicity is becoming common in HIV patients treated with TDF. Numerous case reports and case series have described severe cases of renal tubular toxicity associated with TDF exposure [3-5]. It is estimated that renal tubular dysfunction develops in nearly 15% of patients treated with tenofovir for 2–9 years [6]. Tubular dysfunction may precede the decline of renal function [7].

The main site of TDF toxicity is the proximal tubule, and in severe cases, patients can develop Fanconi syndrome (which is characterized by phosphaturia, glycosuria, bicarbonate wasting , tubular proteinuria, and aminoaciduria,) or acute kidney injury [7]. Several case reports, observational studies, and animal models, support the view that tenofovir is a proximal tubular toxin [8-10].

Current evidence suggests that mitochondria are the subcellular target organelles of tenofovir. Several human and animal studies have shown damage to specifically renal proximal tubular mitochondria [11-13]. In the TDF treated HIV patients who underwent kidney biopsy, the main abnormality on light microscopy was acute proximal tubule damage, and the presence of intracytoplasmic inclusions. Electron microscopy showed widespread morphologic abnormalities in proximal tubule mitochondria, with marked variations in size and shape , disruption of cristae, mitochondrial swelling, and intra-mitochondrial deposits [7,14].

It is well known that mitochondrial damage can result in the overproduction of reactive oxygen species (ROS) and reactive nitrogen species (RNS), which upon accumulation can cause oxidative and nitrosative damage to the lipids, proteins and DNA [15,16]. Proximal tubular cells have a requirement for ATP for the active reabsorption of filtered nutrients and ions. Thus, damage to the proximal tubular mitochondria can have two consequences, proximal tubular dysfunction resulting in Fanconi Syndrome and increased production of ROS thereby resulting in increased oxidant stress.

Oxidative stress is inseparably linked to mitochondrial dysfunction, as mitochondria are both generators of and targets for reactive species [17]. Because of the deleterious effects of ROS, the cell is equipped with several antioxidants and antioxidant enzyme systems to detoxify ROS produced [18,19]. Oxidative stress is reported to occur in tissues only after the antioxidant (AO) defense mechanisms are depleted, leaving ROS to attack the cellular macromolecules such as lipids, proteins, and DNA.

Lipid peroxidation and protein carbonyl content are two important parameters to assess the oxidative damage to lipids (and other constituents) and proteins, respectively. Protein carbonyl groups represent an irreversible protein modification, often leading to the inactivation of the proteins [20]. Protein carbonyl content (Pco) is reported to be a sensitive and early marker of oxidative stress to tissues as compared with TBARS [21].

Superoxide anion is the principal ROS produced by the mitochondria mainly from electron transport chain (ETC.). Superoxide Dismutase (SOD) is first line of defense against superoxide which is the principal ROS produced by the mitochondria and it catalyzes the dismutation of superoxide radicals to hydrogen peroxide and molecular oxygen [22]. Glutathione peroxidase, peroxiredoxins, and catalase decompose hydrogen peroxide generated by SOD to water. The antioxidants within the mitochondria include reduced glutathione, glutathione reductase, and carbonic anhydrase. Mitochondrial glutathione is considered as the key survival antioxidant [23].

Overproduction of superoxide anion or deficiency of SOD can result in the mitochondrial accumulation of highly reactive superoxide radicals, which when present in excess can react with mitochondrial nitric oxide to produce peroxynitrite, a reactive nitrogen species (RNS) [24] and a potent oxidant that can modify proteins to form 3-nitrotyrosine (3 NT) [25]. 3NT content is considered to be an indicator of oxidative modification of proteins along with protein carbonyl content [25].

Therefore, in the present study we determined the effect of chronic TDF administration on proximal tubular mitochondria , parameters of oxido-nitrosative stress , and antioxidant system in the kidneys of rats. The results of the present study show that TDF administration results in severe damage to the proximal tubular mitochondria. Proximal tubular damage was accompanied by increased lipid peroxidation and protein oxidation as well as depletion of the antioxidant system including reduced glutathione (GSH), MnSOD, carbonic anhydrase, glutathione peroxidase, and glutathione reductase. We suggest that TDF induced proximal tubular mitochondrial damage and increased oxidative stress in the kidney may be due to depletion of the antioxidant system, particularly the mitochondrial system.

## Methods

The rat is proven to be a useful model mechanism of nephrotoxicity of a number of agents, including gentamicin, an antibiotic, cisplatin, a chemotherapeutic drug, and acetaminophen, an antipyretic drug. Therefore, we chose the rat model for our study.

### Animals

Adult male Wistar rats (200–250 gm.) were used for the studies. They were housed in standard rat cages (421 × 290 × 190 mm). All animals were exposed to 12 hour light–dark cycles and allowed access *ad libitum* to water and standard rat chow. The proposal was approved by the Institutional review board .The experiments done were approved by the institutional animal ethics committee and were in accordance with the guidelines of the Committee for the Purpose of Control and Supervision of Experimentation on Animals (CPCSEA), Government of India.

### Standardisation of the TDF dose

In order to standardise a model of TDF nephrotoxicity that resembles that of humans, we carried out pilot studies using rats. We first tried the dose that was used by Lebrecht et al. [9]. Rats were treated by oral gavage once a day for 8 weeks with 100 mg kg-1 of TDF. Based on area under the curve exposure, the TDF dose used in this study is about twice the clinical dose (300 mg/day) used in patients [2]. We could not find any renal abnormalities when examined by light microscopy and electron microscopy. Therefore we tried a higher dose that was used by Beisecker et al. [26] i.e. 300 mg/kg body wt. /day, which is 6× the human dose. We could not find any prominent changes in light microscopy and electron microscopy in agreement with that of Beisecker et al. [26],suggesting that a higher dose of TDF is required to produce tubulopathy in rats.

Tenofovir has been shown to cause bone toxicity in animal models, when given 12 times higher dose than recommended for humans [27]. Therefore, we tried 600 mg/kg body wt. /day (corresponds to 12 x human dose). Based on a number of NRTI treatment protocols used by other workers, maximum treatment duration of 5 weeks was used in this study as the duration of treatment for 5 weeks is suggested to model chronic human treatment [28]. Kidneys obtained after chronic 600 mg /kg body wt. TDF treatment for 5 weeks showed proximal tubular damage and dysfunction (manifested as Fanconi Syndrome), severe morphologic abnormalities in proximal tubule mitochondria, such as giant mitochondria, disruption of cristae, mitochondrial swelling, and presence of amorphous deposits in the mitochondrial matrix, the findings that were in close comparison with human kidney biopsies obtained from TDF treated HIV patients. Therefore, we carried out the studies on TDF nephrotoxicity by the administration of 600 mg/kg body wt./d (12 x clinical dose) by gavage for 5 weeks to rats.

### Animal treatment

The rats were assigned randomly into 2 groups and were treated as follow.

Group I (control): The rats in this group (n = 6) received sterile water by gavage.

Group II-The rats (n = 6) in this group received 600 mg/ kg body weight Tenofovir disoproxil fumarate daily by gavage for 5 weeks.

Control animals were administered sterile water by gavage on the same schedule as TDF treatment and were killed at the same time point as that of TDF treated rats.

### Mortality checks, clinical observations, and body weights

Animals were checked daily for clinical signs of toxicity, morbidity, or death. Body weights were measured daily just before gavage.

Twenty-four hours before sacrifice, the rats were placed individually in metabolic cages, and urine was collected for biochemical analysis. Urine samples were centrifuged to remove any suspended material, and the supernatants were used immediately for clinical chemistry. On the 36^th^ day, after overnight fast, blood samples were collected from the rats under halothane anesthesia, by cardiac puncture into tubes and allowed to clot at 20°C. Thereafter, serum was separated by centrifugation at 1200 g for 15 min at 4°C for clinical chemistry. The serum was frozen and the analytes were measured on the third day after sacrifice of the rats.

The animals were then killed by over dose of halothane anesthesia. The abdomen was opened by midline incision and both the kidneys were dissected out, cleaned off the extraneous tissue and weighed. Half of left kidney was cut in cross-section and fixed in 10% buffered formalin for light microscopy, and the remaining half was fixed in 3% glutaraldehyde for electron microscopy. Each right kidney was snap-frozen in liquid nitrogen and stored at - 70°C for subsequent biochemical assays. The biochemical parameters were assayed the following day in freshly prepared homogenates as described later.

### Morphological examination of the kidney

After fixating the kidney tissues in 10% buffered formalin for 24 h at room temperature, the slices were embedded in paraffin and then sectioned. Four micrometer-thick paraffin sections were stained with hematoxylin and eosin for light microscope examination using conventional protocol [29]. A minimum of 8 fields for each kidney section were examined and assigned for severity of changes by an observer blinded to the treatments of the animals. Since the tenofovir-induced morphological abnormalities in kidney are mainly localized to the proximal tubules, and the other structures of the kidney do not exhibit major histological alterations, only the renal cortex was examined in detail.

### Examination of the ultrastructural changes in the kidney tissues by electron microscope (EM)

Electron microscopy was done based on the methods employed routinely in the Lewis lab. [30]. The kidney tissues were fixed in 3% glutaraldehyde and washed in buffer, post fixed by 1% osmium tetraoxide and washed in buffer, and, dehydrated in increasing concentrations of alcohol. The tissues were washed with propylene oxide and embedded in epoxy-resin embedding medium. Sections (0.5 μ) were cut with glass knives and stained with Toluidine Blue for orientation. Ultrathin (900 Å) sections were cut with a diamond knife, stained with uranyl acetate and lead citrate and examined by EM, evaluated and photographed. Each EM photomicrograph was reviewed independently by two investigators. Parameters included presence of structurally abnormal mitochondria, alteration in mitochondrial number, mitochondrial swelling, cristae disruption, and presence of intra-mitochondrial deposits [31]. The extent of mitochondrial ultrastructural injury in the proximal tubular cells was then quantitatively assessed (Scale of 0–5) based upon the cell injury staging system described by Trump and colleagues [31]. Mitochondrial injury scores greater than 3.0 are predictive of ultimate cell death.

### Serum clinical chemistry

Frozen serum was thawed separated out and used for the estimation of phosphate, potassium, bicarbonate, uric acid, glucose, urea and creatinine by conventional spectrophotometric methods.

### Urinalysis

Signs, and symptoms associated with mitochondrial dysfunction in proximal tubular cells include phosphaturia, bicarbonate wasting, tubular proteinuria, glycosuria, and aminoaciduria - acquired Fanconi Syndrome [9]. Urine samples were centrifuged to remove suspended material, and the supernatants were used for the estimation of bicarbonate, phosphate, potassium, and uric acid by standard spectrophotometric methods. Glucose and protein were semi quantified by dipstick. Low molecular weight proteins in urine were detected by SDS PAGE.

### Detection of low molecular weight proteins in urine by SDS PAGE

Urine proteins were measured by Lowry’s method and fractionated by SDS-PAGE using 8% resolving gel and 5% stacking gel [32]. Each sample containing 100 μg of urinary protein was mixed with protein dissociation buffer in the ratio of 1:1 and kept in a boiling water bath for 5 mins. Samples were briefly centrifuged; they were then loaded onto wells. Running gel buffer (pH 8.6) was added to electrophoresis tank. The apparatus was connected to the power pack and was run at 70 V till the sample reached the separating gel. The voltage applied was increased to 90 V at this point. Electrophoresis was stopped when the marker dye reached near the end of the gel. After electrophoretic separation, the gel was stained with Coomassie blue solution (0.01% Coomassie brilliant blue R 250, 50% (v/v) methanol and10% (v/v) glacial acetic acid) for 3 hrs. at room temperature and subsequently destained in the destaining solution (50% (v/v) methanol and 10% (v/v) acetic acid) for 2 h. The gel image was captured and analysed by a gel documentation system (Alpha Innotech).

### Immunohistochemical localization of nitrotyrosine in kidney

Nitrotyrosine was detected immunohistochemically as described by Cuzzocrea et al. [33]. The kidney tissue was fixed in 10% formalin, 4 μ thick sections obtained from paraffin-embedded tissues. After deparafinization, the sections were permeabilized with 0.1% Triton X-100 in Tris buffered saline for 15 min. The primary monoclonal antinitrotyrosine antibody, designated 39B6, raised against-(4-hydroxy-3-nitrophenylacetamido) propionic acid–bovine serum albumin conjugate was obtained from Santa Cruz and the Super Sensitive Polymer/HRP (horse radish peroxidase)/DAB ( 3,3′-diaminobenzidine) kit was obtained from BioGenex were used. Endogenous hydrogen peroxidase was quenched by 3% hydrogen peroxide. After the buffer wash, the universal protein blocking agent was applied over the sections. Then the primary antibody was applied over the sections and incubated overnight. The bound primary antibody was detected by the addition of secondary antibody conjugated with horseradish peroxidase polymer and DAB substrate. After that the slides were counterstained with Harris hematoxylin and mounted.

### Biochemical studies

Biochemical studies were carried out on 10% (w/v) of kidney homogenates prepared in ice-cold 1.5% KCl in a Potter-Elvehjem homogenizer. The homogenate was centrifuged at 4°C at 11,000 g for 30 min to remove unlysed particles. The supernatant was used for the assays.

#### Thiobarbituric acid reacting substances (TBARS)

TBARS was measured as describe by Ohkawa et al. [34]. The mixture consisted of 0.8 ml of sample homogenate (1 mg protein), 0.2 ml of 8.1% SDS (Sodium Dodecyl Sulphate), 1.5 ml of 20% glacial acetic acid adjusted to pH 3.5, and 1.5 ml of 0.8% aqueous solution of TBA (thiobarbituric acid). The mixture was made up to 4 ml with distilled water and heated at 95°C for 60 min. using a glass ball as condenser. After cooling with tap water, 1 ml distilled water and 5 ml n-butanol and pyridine mixture (15:1) were added and the solution was shaken vigorously. After centrifugation at 2000 g for 10 minutes the absorbance of the organic layer was measured at 532 nm. Amount of thiobarbituric reacting substances formed is calculated from standard curve prepared using malondialdehyde, the hydrolytic product of 1, 1′, 3, 3′ tetramethoxy propane and the values expressed as nmoles per mg protein.

#### Protein carbonyl content

Protein carbonyl content was measured using dinitrophenyl hydrazine (DNPH) as described by Sohal et al. [35]. To 0.5 ml of sample (1–2 mg), an equal volume of 10 mM DNPH in 2 N HCl was added and incubated for 1 hr. shaking intermittently at room temperature. Corresponding blank was carried out by adding only 2 N HCl to the sample. After incubation, the mixture was precipitated with 10% TCA (final concentration) and centrifuged. The precipitate was washed twice with ethanol: ethyl acetate (1:1) and finally dissolved in 1 ml of 6 M guanidine HCl, centrifuged at low speed and the supernatant was read at 366 nm. The difference in absorbance between the DNPH treated and HCl treated sample is determined and expressed as nmoles of carbonyl groups per mg of protein, using extinction coefficient of 22 mM^-1^ cm^-1^.

#### Assay of anti-oxidant enzyme activities

##### Superoxide dismutase

Superoxide dismutase was measured as described by Ohkuma et al. [36]. Mixture reaction contained in a final concentration: 0.122 mM EDTA, 30.6 μM MTT (3-(4, 5-dimethythiazol-.2-yl)-2, 5-diphenyl tetrazolium bromide), 0.122 mM xanthine, 0.006% bovine serum albumin, and 49 mM sodium carbonate. Five hundred μL (50–150 μg protein) of tissue homogenate, was added to 1.66 mL of the mixture described above, then 50 μL xanthine oxidase, in a final concentration of 2.8 U/L, was added and incubated in a water bath at 27°C for 30 min. The reaction was stopped by the addition of 0.66 mL of 0.8 mM cupric chloride and the optical density was read at 540 nm. Amount of superoxide formed is calculated using the molar extinction coefficient of MTT (3-(4, 5-dimethythiazol-.2-yl)-2, 5-diphenyl tetrazolium bromide) formazan E 540 of 17,000 M^-1^ cm^-1^ at pH 7.4 to 10.5. The percentage of inhibition by the presence of SOD ( in the tissue sample) is calculated from the reduction of the MTT colour formation as compared to the MTT formazan formed in the absence of SOD (sample replaced by distilled water), which is taken as 100% . One unit of SOD is defined as the amount of protein required to inhibit MTT reduction by 50%. Results were expressed as U/mg protein.

##### Glutathione reductase

In the presence of enzyme, hydrogen is transferred from NADPH to GSSG and the reaction can be measured at 340 nm [37]. To the reaction mixture containing 0.05 ml of 1 M phosphate buffer pH 7.6, 0.15 ml of 10 mM EDTA, 0.1 ml of 1 mM NADPH, and 0.1 ml 10 mM GSSG, the homogenate was added. The volume was made up to 1 ml and the decrease in OD at 340 nm was measured for 2–3 min. One unit is the amount of enzyme needed to oxidise 1 μmole of NADPH/min.

##### Glutathione peroxidase

Total peroxidase is determined by following the oxidation of NADPH at 340 nm using hydrogen peroxide [38]. To 0.25 ml of 0.4 M phosphate buffer, 0.2 ml of 4 mM EDTA, 0.2 ml of 10 mM GSH, 0.2 ml of NaN_3_, 0.2 ml of 1.6 mM NADPH, 0.03 ml glutathione reductase (one unit) and the sample were added. Total volume was made up to 2 ml with water. Reaction was started by adding 0.2 ml of H_2_O_2_ and change in OD at 340 nm was followed. Extinction coefficient of 6.22 × 10^3^ M^−1^ cm^−1.^was used for the calculation. One unit is the amount needed to oxidize 1 nmole of NADPH/min.

##### Carbonic anhydrase (CA)

Carbonic anhydrase activity is measured spectrophotometrically based on the action of enzyme on P-nitrophenyl acetate (Sigma protocol). To 0.2 ml of 3 mM p-nitrophenyl acetate 15 mM tris buffer pH 7.6 was added and made up to 1.0 ml. The reaction was started by the addition of 10 μl sample. It was mixed and change in OD measured at 348 nm for 3 min. One unit of enzyme is the amount required to form one micromole of p-nitrophenol in one minute.

#### Nonprotein thiol (glutathione)

Nonprotein thiol was determined by the method described by Sedlak and Lindsay [39]. Briefly, proteins were removed by the addition of 21 μl 50% trichloroacetic acid (TCA) to 200 μl of sample. Then, samples were centrifuged at 12000 rpm for 10 minutes. 50 μl obtained TCA extract and 100 μl 6 mmol/l dithionitrobenzene (DTNB) (Ellman’s reagent) were added successively to 850 μl 0.2 mmol/l phosphate buffer, pH 8.2, and after 1 hour the absorbance was measured at 412 nm. The results were read from a standard curve prepared from 1 mmol/l solution of reduced glutathione

#### Assay of renal nitrate content

Total nitrate content in the kidney was determined by the method described by Sastry et al [40].To 100 μl of homogenate, 400 μl of Carbonate buffer and 0.15 g of cadmium filings were added and incubated at room temperature for 1 hour with thorough shaking. The reaction was stopped by adding 100 μl of 0.35 M NaOH and 120 mM ZnSO4 and incubated at room temperature for 10 minutes and centrifuged at 4000 g for 10 minutes. 100 μl of clear supernatant was aliquoted into microtitre plate wells and 50 μl of 1% sulfanilamide and 50 μl of 0.1% N–naphthylethylene diamine were added. The contents were mixed well and the color was read at 545 nm spectrophotometrically after 10 minutes.

#### Assay of succinate dehydrogenase

Activity of succinate dehydrogenase was assayed using 2-(p-iodophenyl)-3-(p-nitrophenyl)-5-phenyl tetrazolium (INT) as electron acceptor, which forms formazan on reduction [41]. To 0.1 ml of potassium phosphate buffer, 0.1 ml of INT, 0.1 ml of sodium succinate and sample homogenate were added and volume was made up to 1 ml with water. The assay mixture was incubated in 37°C water bath for 15 minutes. After incubation, reaction was arrested with 10% TCA and the formazan formed was extracted into 4 ml of ethyl acetate and this was read at 490 nm spectrophotometrically. Molar extinction co – efficient of formazan in ethyl acetate is 20.1 x 10^3^ at A 490 nm. One unit of enzyme is defined as the amount required for converting 1 μmol of INT to its formazan per minute under standard assay conditions.

#### Protein content

The protein content of the homogenate/ supernatant was determined as described by Lowry et al. [42].

### Statistical analysis

The results are expressed as mean ± S.D. Student’s t test was used to compare the means. A P < 0.05 was considered statistically significant.

## Result

### Effect of TDF on body weight and kidney wt. of rats

**Figure 1:**
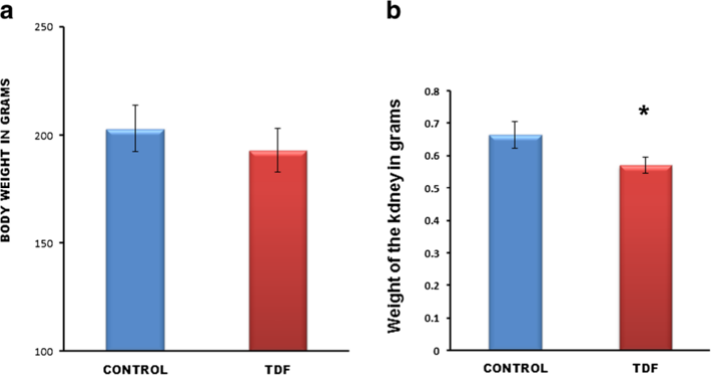
**(a) Body weight and (b) kidney weight of control rats and TDF treated rats.** Significant reduction in kidney weights between control rats and TDF treated rats. Values represent mean ± S.D., n = 6. * P < 0.05 vs. control.

The body weights and kidney weights of control rats and TDF treated rats is shown in Figure 1. There was significant difference in kidney weights between control and TDF treated rats (Figure 1b). The kidney weight was decreased by 9% in the TDF- treated rats as compared with control. The body weight did not show any significant change although it there was a decreasing trend (Figure 1a).

### Effect of TDF on the histology of the rat kidney

**Figure 2:**
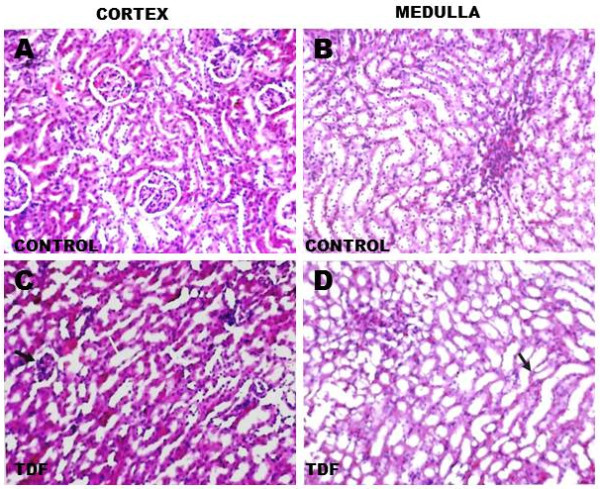
**Representative light micrographs of rat kidney. (A)** Renal cortex of a control rat-shows normal architecture [H& E X 200]. **(B)** Renal medulla of a control rat shows normal architecture [H & E, X 200]. **(C)** Renal cortex of a TDF treated rat. The proximal convoluted tubules were distorted and their lining epithelium was destroyed (white arrow, H & E, X 200). Some glomeruli were shrunken (black arrow). **(D)** Renal medulla of a TDF treated rat–There was mild destruction of the lining epithelium of the loops of Henle and the convoluted tubules (black arrow) H & E, X 200.

The histology of kidneys of control rats and TDF treated rats is shown in Figure 2. The kidneys of control rats showed normal morphology. In the cortex normal glomerular and tubular components (A) were seen and in the medulla normal renal tubular elements were seen (B).

TDF induced renal damage involved mainly the cortex and to a lesser extent, the medulla. The proximal convoluted tubules appeared distorted and their lining epithelium was destroyed. Some glomeruli were shrunken(C).In the medulla there was mild destruction of the lining epithelium of the loops of Henle and the collecting duct (D).

### Ultra structural changes in the rat kidney after TDF treatment

**Figure 3:**
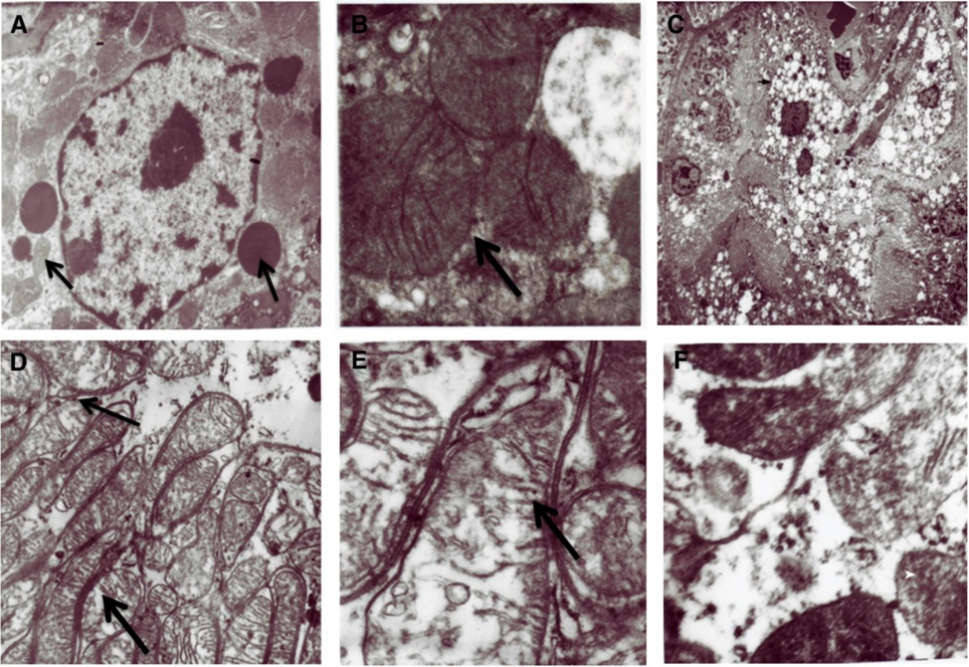
**Representative electron micrographs of control kidney and TDF treated kidney**. **Control kidneys A &B. (A)** Normal Kidney tubules (original magnification × 22000). **(B)** Normal mitochondrial structure (black arrow) in the renal tubules of control rats (original magnification × 22000) .**C- F.** Representative electron micrographs of TDF treated rat kidney. (C) Vacuoles seen in the cytoplasm of the kidney tubule (black arrow) Less number of lysosomes (white arrow) **(D)** Swollen mitochondria (M) black arrow(original magnification × 22 000) **(E)**. Disruption of mitochondrial cristae (black arrow) in the renal tubules of TDF treated rats **(F)** Amorphous deposits in the mitochondrial matrix (white arrow)x 22,000.

Figure 3 shows the ultrastructural findings in the control rats and TDF treated rats. Vehicle-treated rats showed normal tubular structure with numerous mitochondria and lysosomes (black arrows) (A). Oval mitochondria with densely packed cristae were observed (B). Proximal tubular epithelia of TDF-treated rats showed moderate to severe damage to the mitochondria. The cytoplasm showed increased number of vacuoles and reduced number of lysosomes. Nucleus appeared shrunken (C). The mitochondria showed marked variations in size and shape. Mitochondrial toxicity included swollen (giant) mitochondria (D), disrupted cristae (E), and accumulation of amorphous deposits in the mitochondrial matrix (F). An increase in the number of mitochondria with irregular shape and fragmented cristae was observed in the cytoplasm of basal part of tubule cell. In some epithelial cells, the mitochondria were reduced in number. Mitochondrial injury score which was quantitatively assessed (Scale of 0–5) based upon the cell injury staging system described by Trump and colleagues showed a score of 4 or 5.This corresponds to stage V of cellular injury as mitochondrial injury scores greater than 3.0 are predictive of ultimate cell death [31].

### Effect of TDF on proximal tubular function tests

Proximal tubular function was impaired in TDF-treated rats, as evidenced by increased urinary phosphate, potassium and bicarbonate excretion and a considerable reduction in serum phosphate and potassium (Table 1). Tubular proteinuria was also detectable by SDS PAGE. Dipstick method for glucose and protein detection was negative.

**Table 1:** Serum and urine levels of potassium, phosphate, bicarbonate, uric acid, and glucose in the TDF treated rats and control rats

**Parameter**	**Control**	**Test**
	**(n = 6)**	**(n = 6)**
**Serum**		
Phosphate ( mmol/L)	5.88 ± 0.78	4.10 ± 0.36*
Potassium (mmol/L)	6.31 ± 0.47	5.58 ± 0.51*
Uric Acid (mg%)	1.53 ± 0.35	2.9 ± 1.3
Glucose (mg%)	94.33 ± 12.6	90.8 ± 15.4
**Urine**		
Bicarbonate (mmol/L)	0.25 ± 0.058	2.56 ± 1.21**
Uric acid (mg/100 mL)	5.1 ± 1.69	12.9 ± 5.0**
Potassium ( mmol/L)	2.68 ± 1.3	10.38 ± 2.9**
Phosphate ( mmol/L)	0.27 ± 0.1	4.68 ± 1.52*

Values are represented as Mean ± S.D., n = number of rats. * P < 0.05, ** P < 0.01 vs. control.

### Effect of TDF on tubular proteinuria

**Figure 4:**
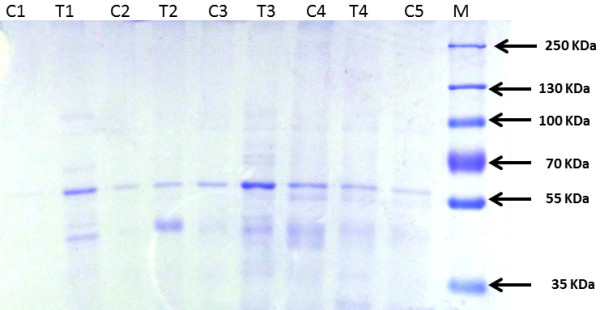
**Urine protein separation by SDS-PAGE electrophoresis.** The urinary protein pattern in the TDF treated rats revealed in addition to band corresponding to albumin, multiple protein bands corresponding to molecular weights less than 55,000 dalton (especially β-2-microglobulin, suggesting complete tubular proteinuria.

The urine protein separated by SDS electrophoresis is shown in Figure 4. This is an useful technique as it identifies low molecular proteins i.e., tubular proteins with mol. wt. less than 55, 000 using a molecular weight marker protein. In SDS-PAGE proteins are separated based on their molecular weight. Individual proteins can then be identified within these patterns.

Urine from normal rats when subjected to electrophoresis yielded an identifiable protein band that corresponded to approximately Mr. Wt. 60, 000, suggestive of albumin. In addition, faint bands were also observed in some controls corresponding to Mr. Weight less than 55KDa. . Alpha1 microglobulin is also detectable in normal urine .Thus, normal rats appear to excrete small amounts of albumin that are detectable only by SDS-PAGE electrophoresis (and not by dipstick), and negligible amount of low molecular weight tubular proteins in urine.

Tubular proteinuria is characterized by the excretion of low-molecular-weight proteins such as alpha 1-microglobulin or retinol-binding protein (RBP) predominantly and is reported to correlate better with the extent of tubulo-interstitial damage than does the determination of total 24-h protein levels. The urine protein pattern in the TDF treated rats revealed at least two bands of molecular weight lesser than 55KDa (in addition to albumin), suggesting tubular dysfunction.

### Renal nitrate content

**Figure 5:**
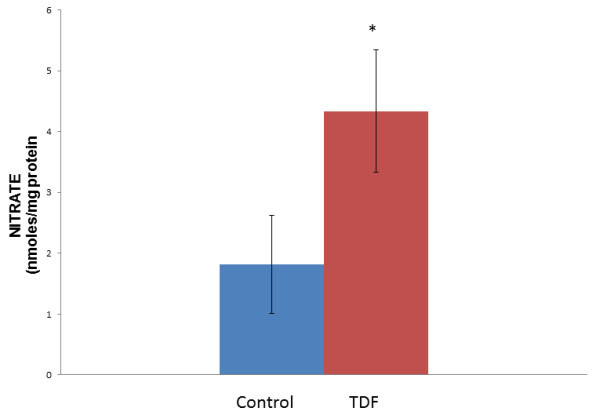
**Nitrate content in the kidney of control rats and TDF treated rats.** Data represent mean ± SD, n = 6 in each group,*p < 0.05 compared with controls.

Figure 5 shows the renal content of nitrate. More than 2.5 fold increase in nitrate level was observed in the kidneys of TDF treated rats as compared with control.

### Immunohistochemical staining for NT

**Figure 6:**
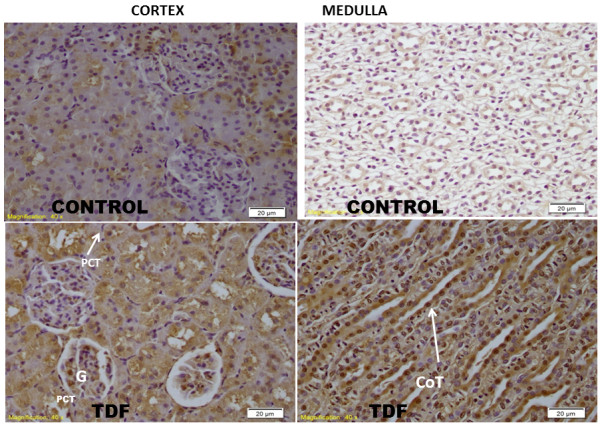
**Immunostaining for nitrotyrosine (NT).** Representative nitrotyrosine staining in the kidneys of rats .In the control rat nitrotyrosine staining was minimal. In TDF treated rat kidney cortex, both proximal convoluted tubule (PCT) and distal convoluted tubules (DCT) stained strongly for NT. The glomerulus (G) showed mild staining for NT. In the medulla, loop of Henle and collecting tubules (CoT) stained for NT (X40).

Immunostaining for NT is shown in Figure 6. NT is considered to be a reliable marker of protein oxidation in addition to protein carbonyl content. Nitrotyrosine immunostaining in the kidneys of control rat was minimal. Glomerulus and the tubules showed mild staining for NT. In the TDF treated rat kidney cortex, both proximal convoluted tubules (PCT) and distal convoluted tubules (DCT) stained strongly for NT. In the medulla, loop of Henle and collecting tubules (CoT) stained for NT.

### Biochemical parameters

**Figure 7:**
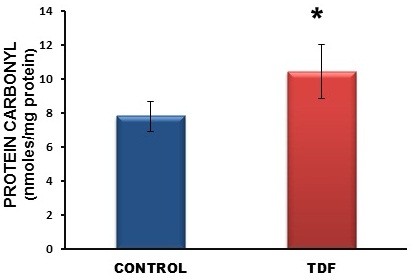
**Protein carbonyl content in the kidneys of control rats and TDF treated rats.** Data represent mean ± SD, n = 6 in each group,*p < 0.05 compared with controls.

**Figure 8:**
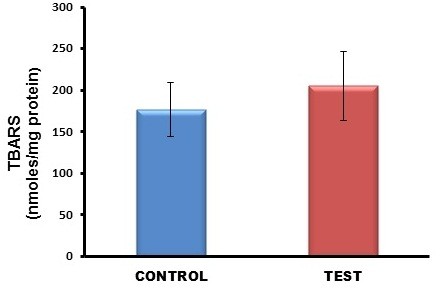
**TBARS levels in the kidneys of control rats and TDF treated rats.** Data represent mean ± SD, n = 6 rats in each group.

**Figure 9:**
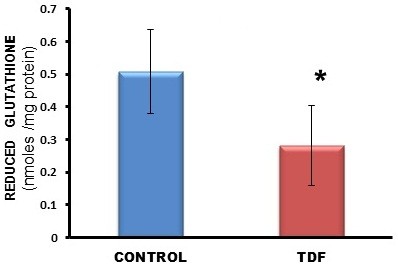
**Reduced glutathione levels in the kidneys of control rats and TDF treated rats.** Data represent mean ± SD, n = 6 in each group , * p < 0.05 compared with controls.

A 50% increase in protein carbonyl content was observed in the kidneys of TDF treated rats as compared with the control (Figure 7). TBARS, an indicator of lipid peroxidation was increased in the kidneys of TDF treated rats as compared with the control, but the increase was not statistically significant (Figure 8). Reduced glutathione, an important intracellular antioxidant was decreased by 50% in the kidneys of TDF treated rats as compared with the control (Figure 9).

**Figure 10:**
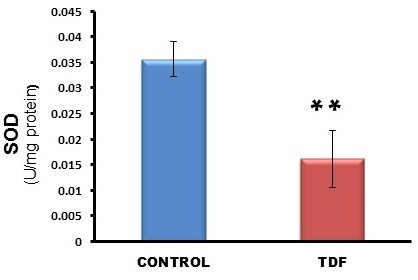
**Superoxide dismutase activity in the kidneys of control rats and TDF treated rats.** Data represent mean ± SD, n = 6 in each group, ** p < 0.01 compared with controls.

**Figure 11:**
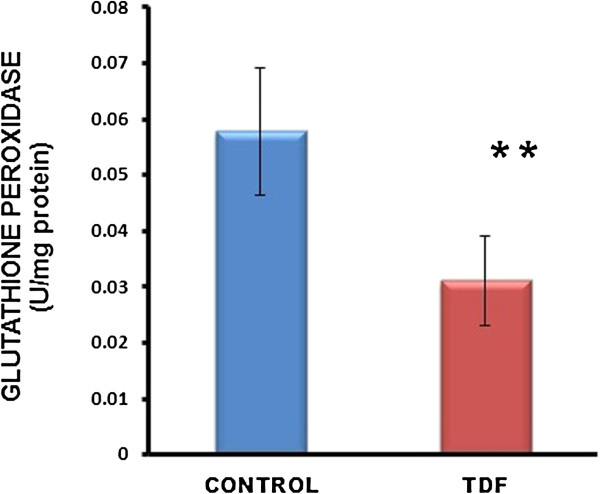
**Glutathione Peroxidase activity in the kidneys of control rats and TDF treated rats.** Data represent mean ± SD, n = 6 in each group .** p < 0.005 compared with controls.

**Figure 12:**
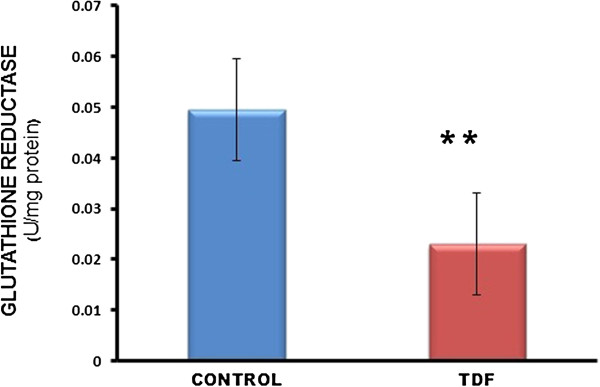
**Glutathione Reductase activity in the kidneys of control and TDF treated rats.** Data represent mean ± SD, n = 6 in each group. ** p <0.005 compared with controls.

**Figure 13:**
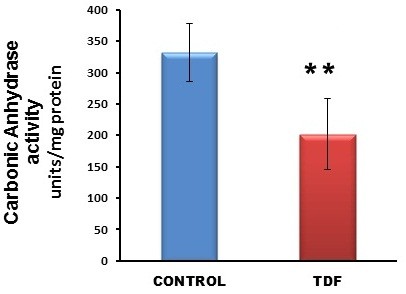
**Carbonic anhydrase activity in the kidneys of control rats and TDF treated rats.** Data represent mean ± SD, n = 6 in each group, ** p < 0.01 compared with controls.

**Figure 14:**
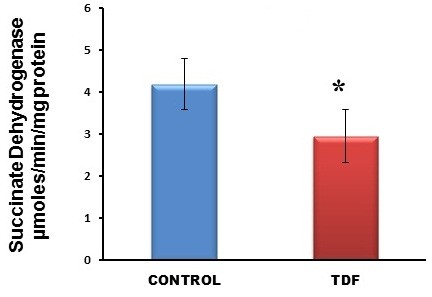
**Succinate dehydrogenase activity in the kidneys of control rats and TDF treated rats.** Data represent mean ± SD, n = 6 in each group,* p < 0.05 compared with controls.

The activities of all the mitochondrial antioxidant enzymes estimated except catalase were significantly decreased in the kidneys of TDF treated rats as compared with the control. The activity of superoxide dismutase (SOD) was decreased by 57% (Figure 10), glutathione peroxidase by 45%(Figure 11), and glutathione reductase by 150% (Figure 12) as compared with control. Carbonic Anhydrase activity was decreased by 45% in the TDF treated rat kidneys as compared with control (Figure 13). Succinate dehydrogenase activity, an indicator of mitochondrial activity was decreased by 29% in the TDF treated rat kidneys as compared with controls (Figure 14), suggesting mitochondrial dysfunction.

## Discussion

Several recent studies have revealed the nephrotoxicity of tenofovir [3-9,18]. Several human and animal studies have shown that TDF is toxic specifically to the proximal tubules, resulting in Fanconi syndrome, characterized by bicarbonate wasting, phosphaturia, amino aciduria, glycosuria, acidosis, and hypophosphatemia. The mechanism of TDF tubulopathy is not clear, however, some mechanisms have been proposed. They are

1. Accumulation of tenofovir in the proximal tubular cells .The proximal tubule plays an important role in drug excretion from the body. TDF enters proximal tubule cells through organic anion transporters (OAT), [43] and exits via the apical transporter MRP4 (multidrug resistance-associated protein 4) [44]. The organic anion transporters can also transport other NRTI such as didanosine. Thus, interactions between TDF and other drugs that are transported by OAT can occur . Ritonavir another anti-viral drug slows TDF renal clearance in humans [45], and increases TDF plasma concentration [46]. As tenofovir is eliminated by active secretion through the proximal renal tubules via MRP4, impaired or delayed elimination of tenofovir would lead to its accumulation and cytoxicity.

2. Direct toxic effect of TDF on the proximal tubules. Studies have shown that TDF causes direct proximal tubular damage, leading to renal failure [47].

3. Mitochondrial toxicity of TDF. Several studies have shown that TDF targets the mitochondria of the proximal tubules and mitochondrial toxicity has been postulated as the major mechanism of TDF-tubulopathy. The most commonly observed effect of TDF on proximal tubular mitochondria is mtDNA depletion. Decreased mtDNA levels have been found in renal biopsies from patients exposed to TDF [12]. It is suggested that tenofovir accumulation in the proximal tubules and its phosphorylation could create an imbalance in nucleotide pools [48], thereby decreasing the availability of nucleotides for mitochondrial DNA synthesis [49]. However, the precise mechanism by which TDF causes mitochondrial injury is not known.

In the present study light microscopic and electron microscopic examination of the kidneys of TDF treated rats revealed damage to the proximal tubular mitochondria confirming the findings of other workers in humans as well as animal models [10,11]. The mitochondria of TDF treated rat kidney tubules were swollen, cristae were disrupted, and amorphous deposits were observed in the matrix. Mitochondrial swelling is considered to be a characteristic feature of deteriorated function of this organelle. Although long term TDF administration has been shown to cause mitochondrial damage, the precise mechanism by TDF induced mitochondrial damage results in renal tubular damage is not known. Proximal tubular cells contain large numbers of mitochondria and are the most reliant upon oxidative phosphorylation and most susceptible to oxidant-induced apoptosis and mutations [50].

Signs, symptoms, and diseases associated with mitochondrial dysfunction in proximal tubular cells include Fanconi syndrome, which is characterized by tubular proteinuria, aminoaciduria, phosphaturia, glycosuria, and bicarbonate wasting [7]. We found bicarbonate wasting, phosphaturia, kaliuresis, low serum bicarbonate, hypophosphatemia and hyperkalemia suggestive of proximal tubular dysfunction. Tubular proteinuria is characterized by the excretion of low-molecular-weight proteins predominantly α_1_-microglobulin or retinol-binding protein (RBP) [51]. Tubular proteins can be detected by SDS PAGE electrophoresis. We were able to detect a faint band corresponding to approximately Mr. Wt. 60,000 and very faint bands corresponding to molecular weight less than 55KDa in the urine of normal rats. Studies reported earlier show that albumin is detectable by SDS –PAGE in the urine of normal rats [52], and in physiologic conditions, the range of daily urinary protein excretion is typically 40–80 mg/24 h. Albumin represents the main component (30–40%), whereas IgG, light chains, and IgA represent 5–10%, 5%, and 3%, respectively, of urinary proteins. The remainder consists mostly of Tamm Horse fall protein [53]. In the urine sample of TDF treated rats , in addition to an intense band suggestive of albumin, we were able to detect the presence of one or more bands that correspond to molecular weight 55 KDa or lesser, which were more intense than that seen in urine of normal rats. According to Al-Bashir et al. [54] normal urine has an albumin band, which is detectable even at a concentration as low as 8 ng/ml. The presence of protein bands in 30,000-50,000 Dalton range is referred to as partial tubular proteinuria, with α_1_-microglobulin as most the predominant protein. In complete tubular proteinuria numerous low molecular weight protein bands, (mainly β_2−_microglobulin) are found in the 8.000-50.000 Dalton range [54]. The urinary protein pattern in the TDF treated rats revealed multiple protein bands corresponding to molecular weights less than 55,000 Dalton suggesting complete tubular proteinuria.

In order to determine whether oxidative stress plays a role in TDF nephrotoxicity, we determined TBARS content and protein carbonyl (Pco) level the indicators of oxidative damage to lipids and proteins in TDF treated rat kidneys. We observed increase in protein carbonyl content suggesting that oxidative stress may play a role in TDF induced renal damage. We could not find any significant difference in renal TBARS levels between TDF treated rats and control rats. This may be attributed to the assay conditions that were employed by us, as we did not add any antioxidants such as butylated hydroxyl toluene to the reaction medium in order to prevent artifactual TBARS formation. In a recent study, Adaramoye et al. [55] have shown that chronic TDF administration to rats results in increase of renal TBARS content by 102%, suggesting enhanced oxidative damage

ROS-induced oxidative stress alters many cellular processes leading to apoptotic cell death. Therefore, the cells are equipped with antioxidant defense systems to combat the ROS. The cellular defense mechanisms include antioxidants such as reduced glutathione and protein thiol, and antioxidant enzymes such as superoxide dismutase, glutathione peroxidase, glutathione reductase, catalase, and carbonic anhydrase.

Mitochondrial glutathione is considered as the key survival antioxidant and its depletion in tissues has been shown to promote oxidative stress and tissue injury [56]. In the present study, we observed a 50% decrease in the GSH content in the TDF treated rat kidneys. Lowering of the mitochondrial GSH (mtGSH) by substances such as alcohol has been shown to make these organelles more susceptible to oxidative damage, and precedes the development of mitochondrial dysfunctions, such as lipid peroxidation and the impairment of ATP synthesis [56].

The level of reduced GSH in the tissues is determined by the activities of two mitochondrial GSH related anti-oxidant enzymes namely glutathione peroxidase (GPO) that consumes reduced glutathione and glutathione reeducates (GR) that regenerates reduced glutathione (GSH) from oxidized glutathione (GSSG). In the present study, decrease in the activities of GPO and GR was observed in the kidneys of TDF treated rats. These findings can be explained as follows. TDF induced mitochondrial damage results in the overproduction of ROS. Excess ROS generated is detoxified by GPO which used GSH as cofactor and during this process; GSH is oxidized to G-S-S-G. The recycling of GSH is a major mechanism that protects cells against ROS and this process is catalyzed by glutathione reductase. Reduction in GR activity in TDF treated rat kidneys may decrease the availability of reduced GSH which is the cofactor for GPO that detoxifies hydrogen peroxide. The lack of availability of GSH may be responsible for the decreased activity of GPO in the TDF treated rat kidneys. This in turn can result in the accumulation of hydrogen peroxide, thereby rendering the cells to increased oxidative stress and tissue injury. Thus significant decrease in reduced glutathione levels induced by TDF, leads to a reduction of effectiveness of the antioxidant enzyme defense system, thereby sensitizing the cells to reactive oxygen species. It is worthwhile to mention here that the decrease in the activities of GPO and GR may be due to their direct inactivation as both the enzymes are susceptible to the attack of reactive species [57].

With respect to the activity of superoxide dismutase, a significant decrease (57%) was observed in the TDF treated rat kidneys. As SOD protects the mitochondrial lipids, proteins, and DNA from the attack of superoxide [58] it is rightly known as the guardian of the power house. Decrease in SOD activity and hence superoxide accumulation can have several deleterious effects on the cell. Firstly, superoxide anion can inactivate several mitochondrial enzymes including aconitase, and the mitochondrial electron chain complexes I and II due to the presence of iron sulphur clusters in them [59], thereby altering their function. Secondly, superoxide anions have proinflammatory roles, causing lipid peroxidation and oxidation, DNA damage, and recruitment of neutrophils to sites of inflammation [60]. Thirdly, excess superoxide radicals can react with mitochondrial nitric oxide to produce peroxynitrite (PON), a reactive nitrogen species (RNS) which can inactivate several mitochondrial enzymes by the nitration of tyrosine residues [59]. Mitochondrial enzymes that are susceptible to the attack by peroxynitrite include electron transport chain components complexes I, II, III and V, as well as glutathione peroxidase, glutathione reductase, aconitase, and MnSOD [61].

3 nitrotyrosine (3 NT) formation is considered to be footprint of peroxynitrite [62]. Animal and human studies have employed analytical and immunological methods for detection of 3-NT, as a marker of peroxynitrite formation in vivo [33]. In the present study intense immunostaining for NT was observed in the TDF treated rat kidneys particularly in the PCT and , DCT suggesting increased peroxynitrite formation in these areas of the kidney. As SOD can be inactivated by PON, decrease in SOD activity observed in the present study may be due its inactivation by PON. Inactivation of SOD can result in the accumulation of toxic superoxide anion which in turn can react with nitric oxide to form peroxynitrite which is a potent nitrosating agent that can cause direct damage to proteins, lipids, and DNA. Thus, inactivation of SOD could lead to self-amplification of oxidative stress in the tissues progressively enhancing peroxynitrite production and secondary damage.

Carbonic anhydrase (CA) catalyzes the reversible hydration of carbon dioxide (CO_2_) to bicarbonate (HCO_3_^-^) [63]. In the kidneys, the proximal convoluted tubules are reported to show the highest CA activity [64]. Studies show the importance of apical and basolateral membrane CA in mediating bicarbonate and fluid absorption in proximal tubules and of the apical membrane CA activity in mediating H^+^ secretion [65]. There are several evidences that suggest that CA acts as an antioxidant [66]. CA functions as an oxyradical scavenger and thus protects cells from oxidative damage and CA over expressing cells exhibit lower free radical levels. Carbonic anhydrase is reported to be involved in glutathione-mediated anti-oxidant activity [67]. In the present study, TDF treatment resulted in significant loss of CA activity in the kidneys. The decrease in CA activity in the proximal tubules can have two effects. Firstly, it can result in renal tubular acidosis due to defective bicarbonate reabsorption in the proximal tubule and therefore its loss in urine (bicarbonate wasting). Secondly, its decreased activity can increase the susceptibility of the tubules to H_2_O_2_. In the present study, we were able to observe proximal tubular damage, loss of CA activity, and bicarbonate wasting in the TDF treated rats. These observations suggest that bicarbonate wasting may be due to the loss of CA activity. It is worthwhile to mention here that CA is an enzyme that is susceptible to inactivation by ROS and RNS [68]. As in our study we have shown that TDF administration results in increased oxidative stress, nitrosative stress and GSH depletion in the kidneys we propose that the loss of CA activity may be due to its inactivation by ROS /and RNS.

Succinate dehydrogenase (SDH) is a key enzyme in the Krebs cycle the activity level of which shows the degree of the activity of mitochondria [69]. A decrease in succinate dehydrogenase activity indicates loss of inner mitochondrial membrane integrity. SDH functions not only in mitochondrial energy generation, but also has a role in oxygen sensing [70]. Its unique redox properties [71] confer SDH a specific function in superoxide handling. Along with ubiquinone, SDH is a crucial antioxidant enzyme in mitochondria controlling superoxide scavenging activity of the respiratory chain. When succinate-ubiquinone activity is inhibited, electrons that would normally transfer through the SDH -B subunit to the ubiquinone pool are instead transferred to O_2_ directly to produce ROS, mainly superoxide anion [72]. In the present study we observed a significant decrease in SDH activity in the TDF treated rat kidneys. This finding not only suggests loss of inner mitochondrial membrane integrity but also suggests that decreased SDH may contribute to increased ROS production and hence oxidant stress.

In summary, the results of the present study show that long term administration of TDF results in proximal tubular mitochondrial damage and proximal tubular dysfunction which manifest as Fanconi Syndrome. TDF induced proximal tubular damage and mitochondrial damage was accompanied by increase in oxidative stress markers and decrease in the antioxidant system namely reduced GSH, SOD, GPO, GR, and carbonic anhydrase. This suggests that the depletion of cellular anti-oxidants contribute to TDF induced damage to the proximal tubular mitochondria. SOD is the first line of defense against mitochondrially derived ROS (superoxide) and so decrease in MnSOD activity can have deleterious effects on the cell. Therefore, logistically overexpression of MnSOD or administration of SOD mimetics should be beneficial in the prevention of TDF nephrotoxicity. We are currently investigating whether the administration of SOD mimetic - Mn (III) ortho N-butoxyethylpyridyl porphyrin (4.5 mg/kg body wt./day and 9 mg/kg body wt. per day ) protects against TDF nephrotoxicity. This compound was chosen for the study as it is a potent SOD mimetic and is less toxic compared with other SOD mimetics [73].

## Conclusion

Tenofovir- induced mitochondrial damage and increased oxidative stress in the rat kidneys may be due to depletion of the antioxidant system particularly, the glutathione dependent system and MnSOD.

## Competing interests

The authors declare that they have no competing interests.

## Authors’ contributions

PA conceived the study, designed it, and drafted the manuscript. BI carried out the histological studies. HR carried out the biochemical assays and immunohistochemical procedures. All authors read and approved the final manuscript.
